# Evaluating drug withdrawal syndrome risks through food and drug administration adverse event reporting system: a comprehensive disproportionality analysis

**DOI:** 10.3389/fphar.2024.1385651

**Published:** 2024-07-10

**Authors:** Zheng Zhang, Qianzhi Yang, Minghao Chen, Wah Yang, Yuping Wang

**Affiliations:** ^1^ Department of Pharmacy, The First Affiliated Hospital of Jinan University, Guangzhou, Guangdong, China; ^2^ Department of Metabolic and Bariatric Surgery, The First Affiliated Hospital of Jinan University, Guangzhou, Guangdong, China

**Keywords:** withdrawal syndrome, real-world data analysis, Food and Drug Administration Adverse Event Reporting System, adverse drug reaction, pharmacovigilance

## Abstract

**Objective:**

The study aims to identify the drugs associated with drug withdrawal syndrome in the Food and Drug Administration Adverse Event Reporting System (FAERS) and estimate their risks of causing withdrawal syndrome.

**Methods:**

All the data were collected from FAERS from the first quarter of 2004 to the third quarter of 2023. Disproportionality analyses of odds ratio (ROR) and proportional reported ratio were conducted to identify potential adverse effects signal of drug withdrawal syndrome.

**Results:**

A total of 94,370 reports related to withdrawal syndrome from the data. The top 50 drugs with most frequency reported were analyzed, and 29 exhibited a positive signal based on the number of reports. The top three categories of drugs with positive signals included opioids, antidepressant drugs and antianxiety drugs. Other classifications included opioid antagonist, muscle relaxant, antiepileptic drugs, analgesics, hypnotic sedative drugs and antipsychotic drugs.

**Conclusion:**

Our analysis of FAERS data yielded a comprehensive list of drugs associated with withdrawal syndrome. This information is vital for healthcare professionals, including doctors and pharmacists, as it aids in better recognition and management of withdrawal symptoms in patients undergoing treatment with these medications.

## Highlights

1. This study first systematically assesses FAERS data for drug withdrawal risks (2004–2023).
2. Our research uncovers significant drug-withdrawal syndrome correlations.
3. We used proportional reporting ratios to identify withdrawal syndrome risk signals.
4. The study presents vital statistics on drugs commonly linked to withdrawal.
5. This research provides new insights for managing drug withdrawal syndrome.

## 1 Introduction

Withdrawal syndrome is characterized by a range of unpleasant symptoms that emerge when an individual with physical dependence ceases or reduces the intake of a habitually used drug (Hoffman RJ, 2007). This syndrome occurs due to the physiological or psychological dependence that develops over time, leading to adverse reactions when the drug usage is discontinued or decreased. Symptoms of withdrawal syndrome vary widely, from physical discomfort to severe psychological distress, influenced by factors such as drug type, usage duration, dosage, and individual physiological differences. Commonly experienced symptoms include restlessness, rapid heart rate, trembling, high blood pressure, elevated body temperature, headache, insomnia, nausea, stomach cramps, vomiting, perspiration, and confusion (Ciuca Anghel et al., 2023). Managing these symptoms is a critical challenge in reducing drug dependence.

Drugs frequently associated with dependency and withdrawal include antidepressants, non-cancer pain opioids, gabapentinoids, benzodiazepines (BDZs), and Z-drugs (zopiclone, zaleplon, and zolpidem) (Marsden et al., 2019). About 20% of patients on antidepressants for over a month report withdrawal symptoms upon abrupt cessation or significant reduction in dosage (First, 2013). In the United States, prescription opioids are highly abused, with the duration of withdrawal depending on the opioid’s half-life (Srivastava et al., 2020). Dependence on opioids often drives individuals to seek these substances, primarily to alleviate withdrawal symptoms, leading to unsafe usage or abuse (Sherman and Latkin, 2002). Gabapentinoid withdrawal may occur within 12 h to 7 days post-discontinuation (Athavale and Murnion, 2023). Severe withdrawal can trigger symptoms like anxiety, insomnia, muscle spasms, tension, perceptual hypersensitivity, and potentially life-threatening conditions (Lader, 2014). This necessitates effective management strategies to mitigate withdrawal symptoms and prevent the development of tolerance.

The mechanisms and risks of withdrawal syndrome vary among different drugs, highlighting a gap in real-world studies concerning drug-associated withdrawal syndrome. Investigating the withdrawal responses to various drugs is essential for understanding the comprehensive effects and risks of drug treatments. Such comparative analyses can improve clinical medication guidance, ensuring patients’ safe and effective use of drugs.

The Food and Drug Administration Adverse Event Reporting System (FAERS) is a database that monitors drug safety by collecting real-world data on adverse events (AEs) (Sakaeda et al., 2013; Ahdi et al., 2023). It represents the largest globally recognized repository of passively reported adverse drug events, covering a broad spectrum of patient populations. This study aims to identify drugs linked to withdrawal syndrome within the FAERS database and detect potential risk signals of drug-induced withdrawal. The findings are intended to assist healthcare professionals in effectively managing withdrawal syndrome in patients.

## 2 Materials and methods

### 2.1 Data collection

This study extracted drug-related data from the FAERS database, covering the period from the first quarter of 2004 to the third quarter of 2023. FAERS, a spontaneous reporting system, updates its database quarterly and provides public access (http://www.fda.gov/drugs/surveillance/questions-and-answers-fdas-adverse-event-reporting-system-faers). AEs within FAERS are coded using the medical dictionary for regulatory activities (MedDRA). The dataset is organized into seven distinct tables: demographic (DEMO), drug (DRUG), report sources (RPSR), therapy (THER), indication (INDI), reaction (REAC), and outcome (OUTC). Each table offers specific information, from patient demographics to drug reactions and outcomes.

### 2.2 Data cleaning

To eliminate duplicate reports, we employed the FDA’s recommended method. This involved sorting the DEMO table’s PRIMARYID, CASEID, and FDA_DT fields by CASEID, FDA_DT, and PRIMARYID. When multiple reports shared the same CASEID, the report with the latest FDA_DT was retained. If CASEID and FDA_DT were identical among reports, the one with the highest PRIMARYID was preserved.

### 2.3 Statistical analysis

Our focus was on medications reported under the preferred terms (PTs), “drug withdrawal syndrome” and “drug withdrawal syndrome neonatal.” Due to varied drug name formats in the database, we first identified each drug’s generic name. We then matched these names with their corresponding medical subject headings [Mesh] on PubMed (https://www.ncbi.nlm.nih.gov/mesh/). This process helped us to create a comprehensive list of drugs associated with the identified Mesh terms. We conducted a disproportionality analysis on the top 50 most reported drugs to evaluate their safety signals. The disproportionality analysis includes the reporting odds ratio (ROR) and the proportional reporting ratio (PRR) is widely used in assess the relationship between the medications and AEs (Sakaeda et al., 2013; Noguchi et al., 2021; Zou et al., 2023). It is based on a two-by-two contingency table (Table 1). The equations were as follow:

$$\text{ROR}=\text{ad}/b/c,95\%\text{CI}=e^{\ln\left(\text{ROR}\right)\pm 1.96(1/a+1/b+1/c+1/\hat{d})0.5}$$

$$\text{PRR}=a\left(c+d\right)/c/\left(a+b\right),\chi^{2}=\left.[{\left(\text{ad}‐\text{bc}\right)}^{2}\right]\left(a+b+c+d\right)/\left[\left(a+b\right)\left(c+d\right)\left(a+c\right)\left(b+d\right)\right]$$

**TABLE 1:** Disproportionality analysis based on two-by-two contingency table.

	Target adverse events	Other adverse events	Total
Target drug	a	b	a+b
Other drugs	c	d	c+d
Total	a+c	b+d	a+b+c+d

(a, number of reports containing both the target drug and target adverse drug reaction; b, number of reports containing other adverse drug reaction of the target drug; c, number of reports containing the target adverse drug reaction of other drugs; d, number of reports containing other drugs and other adverse drug reactions. 95% CI, 95% confidence interval; *N*, the number of reports; χ^2^, chi-squared).

The criteria for a ROR signal were a minimum of three reports (a value) and a 95% confidence interval (CI) lower limit for the ROR > 1. A PRR signal was indicated by at least three reports (a value) with a PRR ≥ 2 and a variance (χ^2^) ≥ 4. A signal was considered significant if it met the criteria of both ROR and PRR, suggesting a potential link between the medication and the PTs. The R software (version 4.3.2) was utilized for data management, cleaning, extraction, and signal calculation. The graphical representation of the data was created using Microsoft 365 Excel, GraphPad Prism 9, and R software.

## 3 Results

### 3.1 Baseline characteristics

**FIGURE 1:**
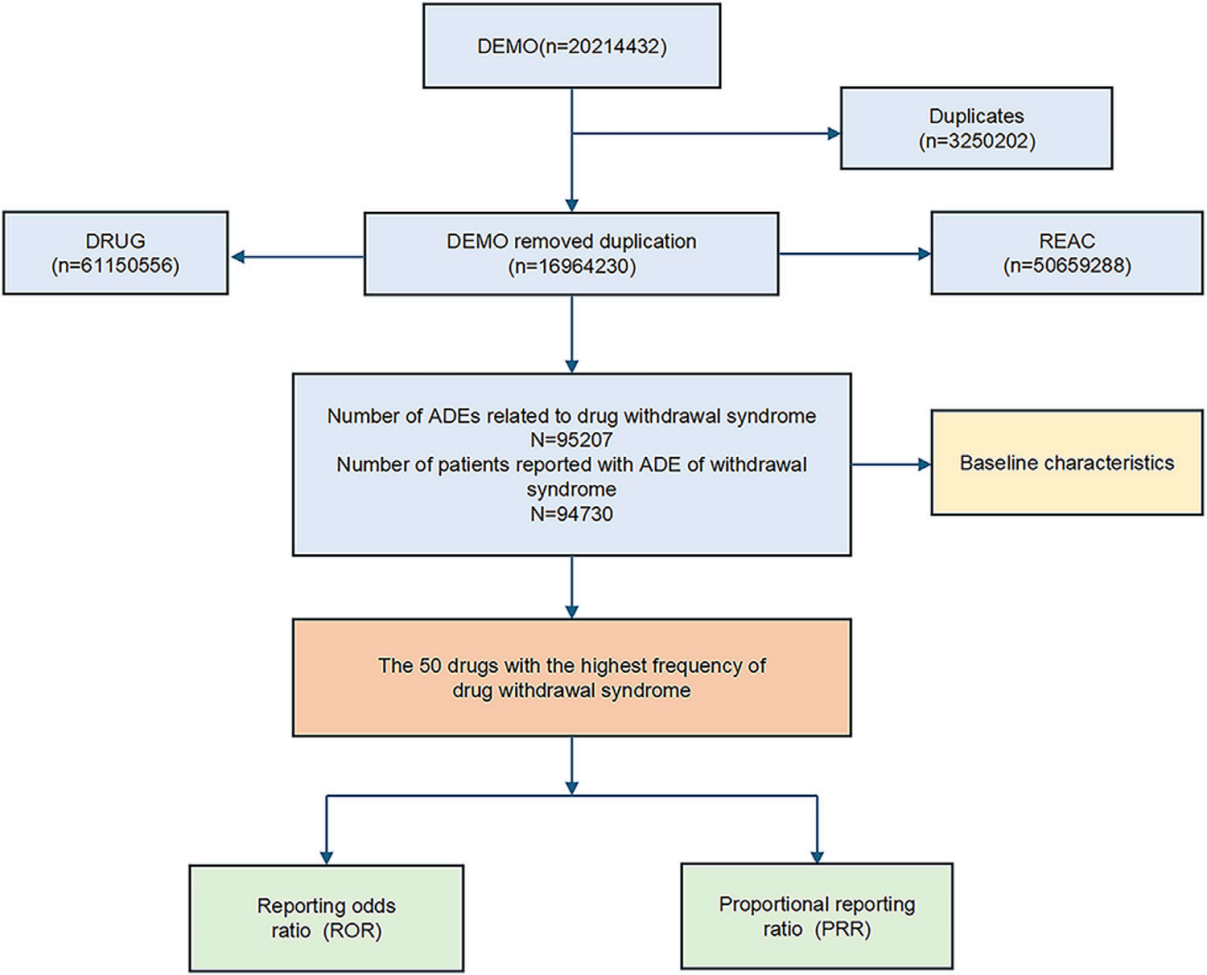
The data mining flow chart of this study.

The dataset spanning from the first quarter of 2004 to the third quarter of 2023 included a comprehensive collection of reports. After removing duplicates, the dataset was refined to include detailed information on drug withdrawal syndrome across 94,756 instances. The data-cleaning process is depicted in Figure 1.

**FIGURE 2:**
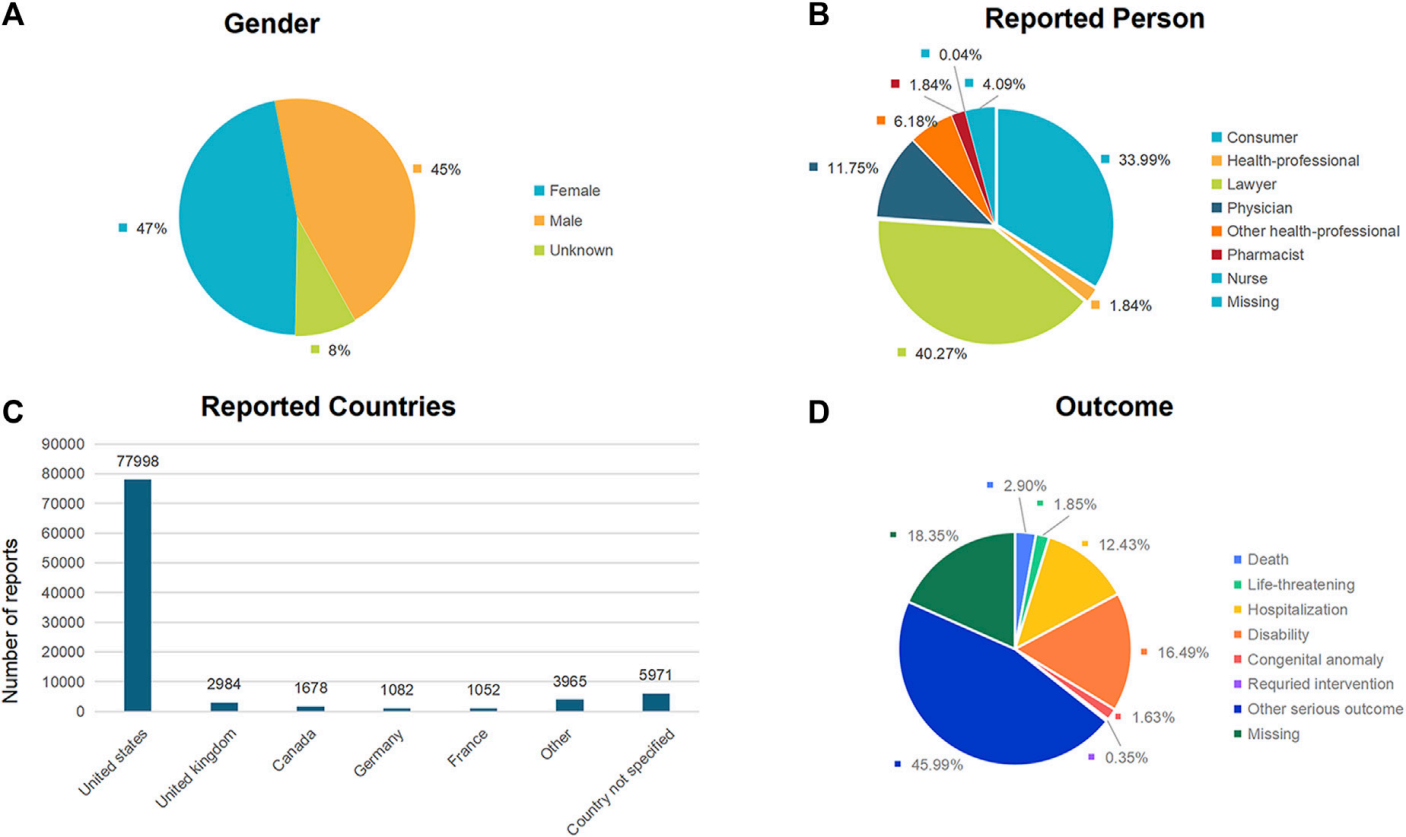
Baseline characteristics of patients and ADEs reports included in this analysis: **(A)** sex distribution of included patients; **(B)** distribution of reporter occupations included in the reports; **(C)** number of reports in different countries; **(D)** outcomes of included patients.

The distribution of reports by gender revealed that 44,188 (46.65%) were female, 42,564 (44.93%) were male, and 7,978 (8.42%) reports had unspecified gender (Figure 2A; Table 2). Excluding the unknown reporters, lawyer and consumer reported the most Aesthetic in 40.27% (n = 38,150) and 33.99% (32,201), respectively (Figure 2B).

**TABLE 2:** Basic patient information.

Characteristics	Number (proportion)
**Gender**	
Female	44188 (46.65%)
Male	42564 (44.93%)
Unknown	7978 (8.42%)
**Reported Person**	
Consumer	32201 (33.99%)
Health-professional	1744 (1.84%)
Lawyer	38150 (40.27%)
Physician	11129 (11.75%)
Other health-professional	5850 (6.18%)
Pharmacist	1740 (1.84%)
Nurse	41 (0.04%)
Missing	3875 (4.09%)
**Outcome**	
Death	2751 (2.90%)
Life-threatening	1754 (1.85%)
Hospitalization	11777 (12.43%)
Disability	15621 (16.49%)
Congenital anomaly	1543 (1.63%)
Requried intervention	328 (0.35%)
Other serious outcome	43570 (45.99%)
Missing	17386 (18.35%)
**Reported Countries (show top five)**	
United states	77998 (82.34%)
United kingdom	2984 (3.15%)
Canada	1678 (1.77%)
Germany	1082 (1.14%)
France	1052 (1.11%)
Other	3965 (4.19%)
Country not specified	5971 (6.30%)

**FIGURE 3:**
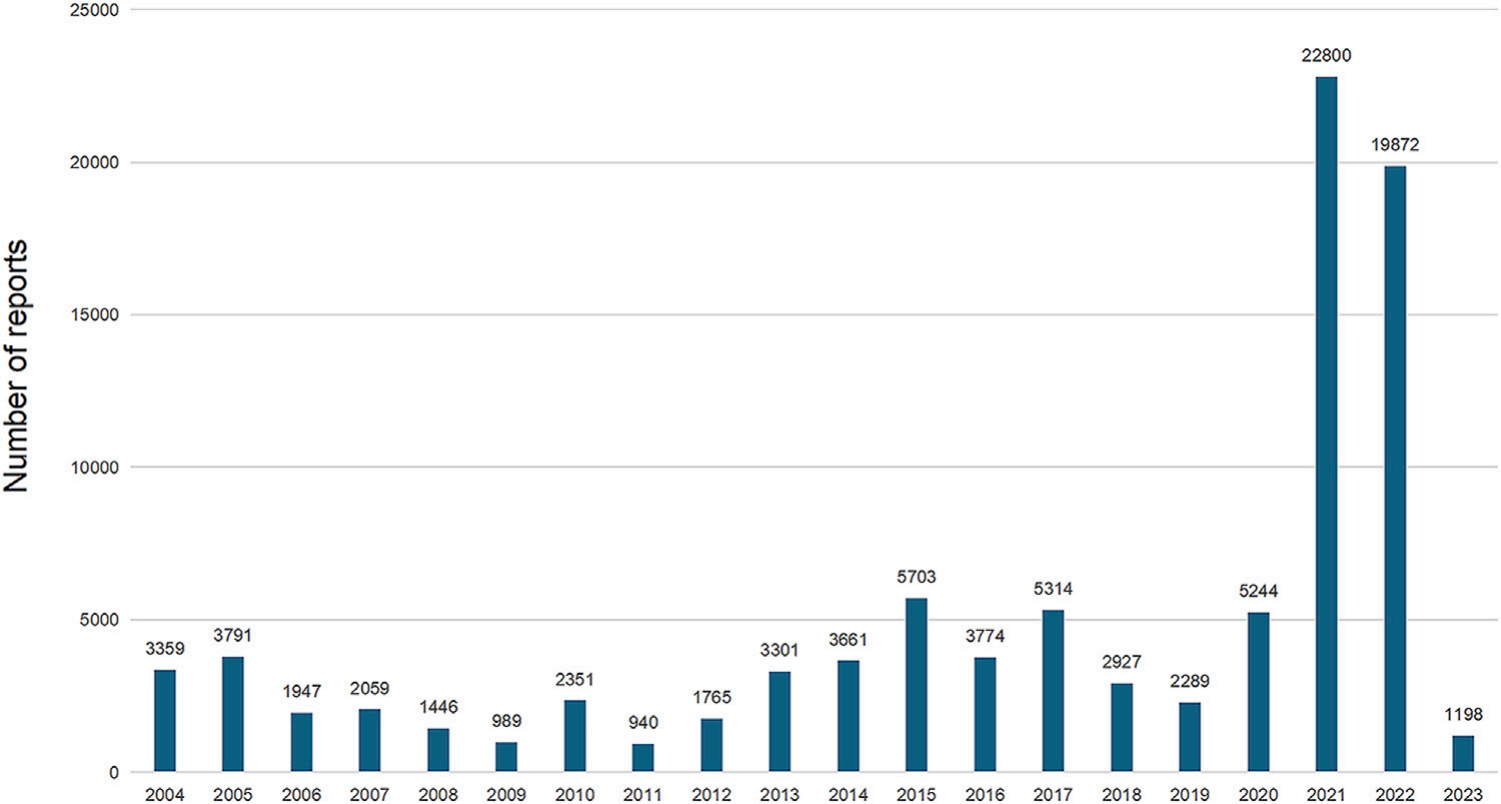
Annual reported withdrawal syndrome cases in the FAERS.

The analysis encompassed 94,730 instances of drug withdrawal syndrome reported from 79 countries and regions. The United States of America accounted for the majority, with 77,998 reports (82.34%), followed by the United Kingdom (2,984 reports, 3.15%), Canada (1,678 reports, 1.77%), Germany (1,082 reports, 1.14%), and France (1,052 reports, 1.11%) (Figure 2C). In cases where a patient had multiple outcomes recorded in the FAERS database, the most severe outcome was prioritized for analysis. Instances where a primary appeared in different years or quarters were treated as distinct events. The most common adverse outcomes identified were classified as “other serious outcomes,” with hospitalization and disability following (Figure 2D). A notable increase in reports was observed in 2021 and 2022, with figures reaching three to four times higher than previous records (Figure 3).

### 3.2 Disproportionality analysis

**FIGURE 4:**
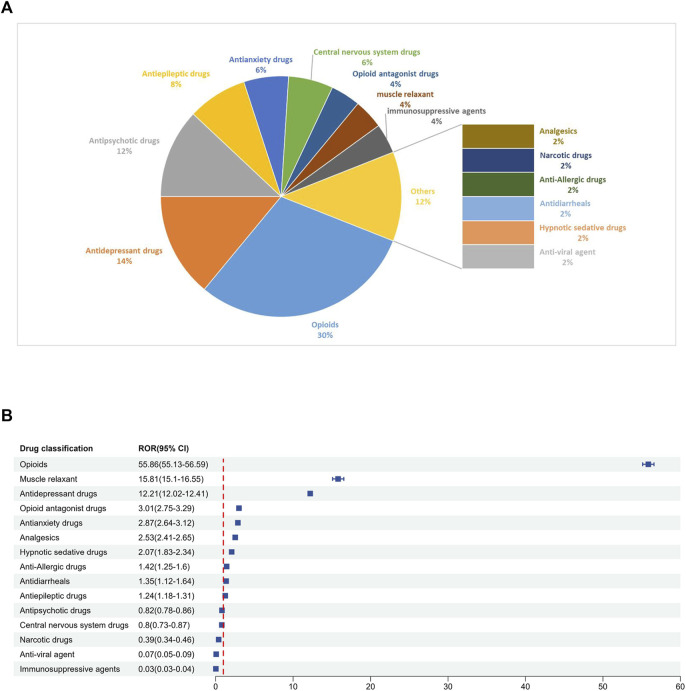
**(A)** Classification of the top 50 reported drugs associated with drug withdrawal syndrome. **(B)** ROR for each classification of the top 50 reported drugs.

Among the 50 drugs most frequently reported (Table 3), the drug classification and distribution were as follows (Figure 4A): opioids (n = 15, 30%), antidepressant drugs (n = 7, 14%), antipsychotic drugs (n = 6, 12%), antiepileptic drugs (n = 4, 8%), antianxiety drugs (n = 3, 6%), central nervous system drugs (n = 3, 6%), opioid antagonist drugs (n = 2, 4%), muscle relaxant (n = 2, 4%), immunosuppressive agents (n = 2, 4%), analgesics (n = 1, 2%), narcotic drugs (n = 1, 2%), anti-allergic drugs (n = 1, 2%), antidiarrheals (n = 1, 2%), hypnotic sedative drugs (n = 1, 2%) and anti-viral agent (n = 1, 2%). Disproportionality analyses were conducted for each drug class. The class of drugs that showed a positive signal included opioids, muscle relaxant, antidepressant drugs, opioid antagonist drugs, antianxiety drugs, analgesics, hypnotic sedative drugs, anti-allergic drugs, antidiarrheals and antiepileptic drugs. The signal values of each drug class are shown in Figure 4B.

**TABLE 3:** Top 50 medications associated with withdrawal syndrome from the FAERS arranged by frequency, 2004Q1 to 2023Q3.

Ranking	Medication	Frequency
1	Oxycodone	37500
2	Duloxetine	7466
3	Paroxetine	6087
4	Buprenorphine	5967
5	Venlafaxine	3586
6	Hydromorhone Hydrochloride	2751
7	Buprenorphine Naloxone	2093
8	Fentanyl	1941
9	Morphine	1913
10	Baclofen	1843
11	Pregabilin	1759
12	Methadone	1116
13	Quetiapine	760
14	Actemainophen Oxycodone	745
15	Hydrococone Acetaminophen	717
16	Gabapentin	688
17	Tramadol	685
18	Aspirin-Oxycodone Hydrochloride- Oxycodone Terephthalate Combination	657
19	Citalopram	585
20	Hydrocodone	476
21	Clonazepam	466
22	Sertraline	364
23	Naltrexone	339
24	Varenicline	325
25	Olanzapine	320
26	Lorazepam	305
27	Fluoxetine	283
28	Diazepam	264
29	Zolpidem	256
30	Cetirizine	254
31	Aripiprazole	241
32	Butorphanol	195
33	Risperidone	178
34	Sodium Oxybate	163
35	Lamotrigine	162
36	Clozapine	156
37	Mirtazapine	156
38	Naloxegol	135
39	Loperamide	109
40	Tapentadol	107
41	Levetiracetam	99
42	Atomoxetine	97
43	Natalizumab	90
44	Lisdexamfetamine Dimesylate	82
45	Ziprasidone	77
46	Hyoscine	70
47	Adalimumab	59
48	Interferon Beta-1A	56
49	Morphine Naltrexone	56
50	Alprazolam	5

There are 29 medications that exhibited positive signals for association with drug withdrawal syndrome. This group included 5 opioid combination preparations and 24 single-drug entities. Opioids emerged as the dominant category, constituting 51.7% of the drugs with positive signals.

According to the ROR signal intensity, the top three opioids were oxycodone [ROR 79.51 (95% CI), (78.46–80.58), PRR (χ^2^), 72.88 (1,620,308.56)], butorphanol [ROR (95% CI), 48.72 (42.10–56.38), PRR (χ^2^), 45.15 (8412.44)]and hydrocholoride [ROR (95% CI), 34.33 (33.01–35.69), PRR (χ^2^), 32.35 (81,318.65)]. Other opioids with positive signals were hydrocodone, methadone, buprenorphine, morphine, fentanyl, tapentadol, tramadol, and five compound preparations: aspirin-oxycodone hydrochloride-oxycodone terephthalate, buprenorphine naloxone, morphine naltrexone, hydrocodone acetaminophen, and acetaminophen oxycodone.

**FIGURE 5:**
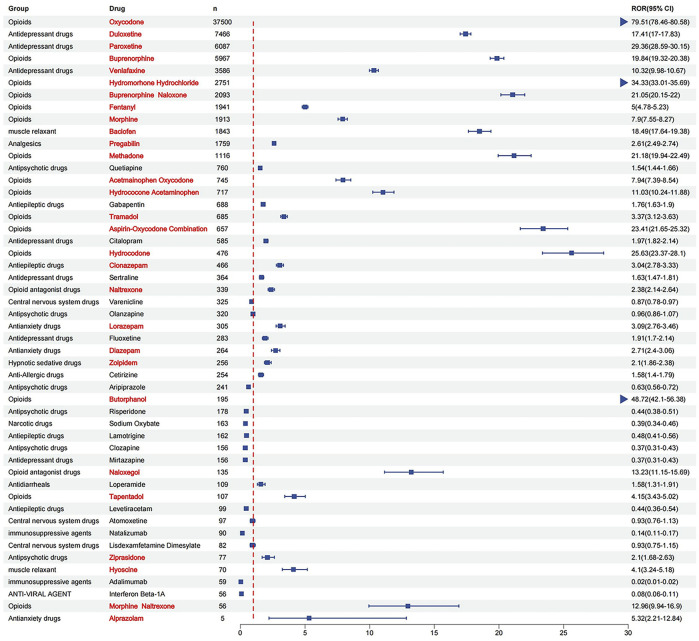
ROR for each of the top 50 drug withdrawal syndrome reports (drug with positive signals were highlighted in red color).

For antidepressants and anxiolytic drugs, three positive signals were identified in each category. The antidepressants with notable signal intensities were paroxetine [ROR (95% CI), 29.36 (28.59–30.15), PRR (χ^2^), 27.95 (148,399.43)], duloxetine [ROR (95% CI), 17.41 (17.00–17.83), PRR (χ^2^), 16.92 (103,338.37)], and venlafaxine [ROR (95% CI), 10.32 (9.98–10.67), PRR (χ^2^), 10.15 (28,520.12)], whereas for anxiolytics, alprazolam, lorazepam, and diazepam were identified. Additionally, positive signals were found for two muscle relaxants and opioid receptor antagonists: baclofen [ROR (95% CI), 18.49 (17.64–19.38), PRR (χ^2^), 17.91 (28,907.81)], hyoscine [ROR (95% CI), 4.10 (3.24–5.18), PRR (χ^2^), 4.07 (162.55)], naloxegol [ROR (95% CI), 13.23 (11.15–15.69), PRR (χ^2^), 12.97 (1490.97)], and naltrexone [ROR (95% CI), 2.38 (2.14–2.64), PRR (χ^2^), 2.37 (268.05)]. The detailed signal values for all drugs are presented in Figure 5 (drug with positive signals were highlighted in red color) and Supplementary Table S1.

## 4 Discussion

This investigation provides a detailed analysis of drug-related withdrawal syndromes using data from the FAERS since its inception in 2004. Our study identified 29 drugs out of the top 50 with the highest report frequencies that showed significant positive signals for withdrawal syndrome based on ROR and PRR analyses. This marks the first endeavor to systematically explore and identify withdrawal syndrome associations with specific drugs within the FAERS database, contributing valuable insights into the risk assessment of drug withdrawal in clinical settings and promoting safer drug use practices.

Withdrawal syndrome’s epidemiology and management, particularly about opioids, antidepressants, and antianxiety drugs, have been the focus of numerous studies. Opioids represent a significant substance-related challenge in the United States, with non-medical use exceeding 90% in 2019 and nearly 20% of users experiencing withdrawal symptoms post-administration (Mannes et al., 2023). Similarly, withdrawal syndrome is a common occurrence during the tapering process of antidepressant medications, notably among those taking selective serotonin reuptake inhibitors (SSRIs) and serotonin-norepinephrine reuptake inhibitors (SNRIs) (Fava et al., 2015, 2018). It is estimated that around 50% of patients who either discontinue or decrease their dose of antidepressant medications experience withdrawal symptoms, with half of these individuals describing the symptoms as severe, according to survey data (Davies and Read, 2019). BDZs are prominently utilized as antianxiety medications in clinical settings (Kelly et al., 2012). Research indicates that approximately 18% of individuals diagnosed with schizophrenia are prescribed antianxiety medications, with a tendency for BDZs to be misused among these patients (Karam et al., 2002; Chakos et al., 2006).

Additionally, withdrawal symptoms have been reported in 30%–100% of individuals undergoing a tapering process of BDZs (Kitajima et al., 2012). Previous research has either concentrated on individual drug classes or omitted a comprehensive assessment of all medications potentially linked to withdrawal syndrome without comparing their risk levels. Hence, leveraging the FAERS database to identify potential risk signals is paramount.

Opioids are commonly prescribed for various types of pain management, including chronic pain related to cancer and non-cancer conditions, as well as for treating opioid use disorder (OUD) (Wiffen et al., 2017; Busse et al., 2018). The onset of opioid withdrawal significantly contributes to the compulsion for patients to continue or seek out opioid prescriptions, with symptoms ranging from insomnia and anxiety to nausea, vomiting, fever, tachycardia, and gastrointestinal disturbances (Weiss et al., 2014; Srivastava et al., 2020). In this study, opioids emerged as the predominant category of drugs associated with positive signals for withdrawal syndrome, accounting for 51.7% of all drugs identified with positive signals. Distinguished by their pharmacokinetics, short-acting opioid agonists (SAOAs), such as morphine, oxycodone, hydrocodone, hydromorphone, and fentanyl, are characterized by rapid onset and short duration of action. Conversely, long-acting opioid agonists (LAOAs) exhibit a slower onset and prolonged effect. The propensity for withdrawal syndrome is higher with SAOAs due to their shorter half-lives and peak plasma concentrations. This results in a significant drop in opioid levels, leading to an imbalance of inhibitory and excitatory neurotransmitters in the brain, thereby precipitating withdrawal symptoms. SAOAs typically induce more immediate and severe withdrawal symptoms compared to the more gradual and milder withdrawal process associated with LAOAs. Within the United States, oxycodone and hydrocodone are the most frequently used opioids, noted for their higher potential for abuse. Therefore, alternative pain management strategies and more effective withdrawal prevention protocols is essential to address the high propensity for opioid dependence and withdrawal.

Furthermore, compound opioids (e.g., aspirin-oxycodone, hydrocodone acetaminophen, and acetaminophen oxycodone) are primarily utilized for moderate to severe pain management, leveraging the synergistic analgesic effects of non-steroidal anti-inflammatory drugs (NSAIDs) and opioids. Compared to monotherapy analgesics, these combination analgesics are favored for their reduced side effects, ease of access, and improved patient compliance (Volkow and McLellan, 2016). Our study also identified positive risk signals for compound opioids, highlighting the potential for addiction and withdrawal symptoms if misused or abruptly discontinued.

Methadone, buprenorphine, and naloxone, which act on opioid receptors, are employed in the treatment of OUD to mitigate overdose risks and the harmful consequences of substance abuse despite maintaining opioid tolerance and physical dependence (Volkow et al., 2019; Torres-Lockhart et al., 2022). A significant observation from our research is the elevated risk of withdrawal syndrome when opioids are combined with opioid receptor antagonists, as opposed to using opioids alone. Buprenorphine, a partial µ-opioid receptor agonist and κ-opioid receptor antagonist (Leander, 1987), along with the buprenorphine + naloxone combination (Suboxone), is approved for opioid addiction treatment. Naloxone is included in the formulation primarily to deter intravenous misuse of buprenorphine. However, if such combinations are administered intravenously by opioid-dependent individuals, they may precipitate withdrawal symptoms. This approach mirrors the combination of morphine with naltrexone sulfate (Embeda), where the rate of withdrawal syndrome AEs in the combined treatment group was higher than in the buprenorphine-only treatment group, consistent with our findings (Fudala et al., 2003). While these combinations aim to reduce intravenous misuse, complete prevention is not achievable, necessitating continuous monitoring of patients on opioid substitution treatment (OST) (Mammen and Bell, 2009).

Antidepressants, widely utilized beyond psychiatric applications, are prescribed for conditions such as obsessive-compulsive disorder (OCD), paranoia, and anxiety. The discontinuation or dosage reduction of these medications can lead to withdrawal symptoms, which may mimic the original disorder or manifest as distinct sensations like electric shock-like feelings, insomnia, and irritability (Henssler et al., 2019). It has been observed that roughly 50% of individuals who cease or lower their antidepressant intake experience withdrawal effects (Sorensen et al., 2022). Such symptoms typically arise within 3 days post-reduction or cessation and can vary in severity, sometimes presenting as new or unexpected. Research has identified that antidepressants with shorter elimination half-lives, including paroxetine and venlafaxine, are more likely to induce withdrawal symptoms (Haddad, 2001; Fava et al., 2015). Analysis of the WHO adverse reaction database revealed that antidepressants such as paroxetine, duloxetine, and venlafaxine were strongly associated with withdrawal symptoms, a finding that corresponds with our research (Gastaldon et al., 2022).

BDZs, often prescribed for anxiety disorders, are known to be frequently misused (Karam et al., 2002). Clinical guidelines recommend limiting the use of BDZs to a few weeks. Yet, it is common for patients, particularly within psychiatric and elderly populations, to use these medications for months or even years. Prolonged use can lead to the development of both psychological and physiological dependencies, making the discontinuation of BDZs challenging and often resulting in withdrawal syndrome (Baandrup et al., 2018). In our analysis, positive withdrawal signals were identified for alprazolam, lorazepam, clonazepam, and diazepam, indicating a significant withdrawal risk associated with these four BDZs. Research indicates that withdrawal symptoms may manifest sooner and with greater severity following the cessation of short-acting BDZs (Noyes et al., 1991). This finding is consistent with the ranking of these drugs in our study. While nBDZs generally present a lower risk of dependence and withdrawal, attention should be directed towards the misuse of Z-drugs, particularly zolpidem, which has been reported in numerous cases of dependence and withdrawal (Wang et al., 2011; Haji Seyed Javadi et al., 2014; Barbosa Eyler and Utria Castro, 2023). Zolpidem was the only nBDZ identified with a positive signal for withdrawal in this study, often in situations involving high doses.

Additionally, our findings identified positive withdrawal signals associated with two muscle relaxants: baclofen and scopolamine. Baclofen is commonly used in the management of conditions such as multiple sclerosis and spinal cord injuries. The most significant complication associated with baclofen therapy is withdrawal syndrome, which can rapidly progress and potentially be fatal (Coffey et al., 2002; Cardoso et al., 2014). This syndrome is particularly prevalent with Intrathecal Baclofen Therapy (ITB) therapy, especially in patients with hepatic and renal dysfunctions, necessitating cautious dose adjustments and vigilant monitoring. Scopolamine patches, used primarily for motion sickness prevention, have been associated with withdrawal symptoms, including exacerbated motion sickness or headaches, as reported in several case studies (Lau and Vaneaton, 2014; Chowdhury et al., 2017; Wahl and Vlad, 2020). These symptoms typically manifest after three or more days of continuous use and are generally self-limiting without leading to severe consequences.

Gabapentin and pregabalin are prescribed for various nervous system disorders including epilepsy and pain syndromes, pregabalin also indicated for generalized anxiety disorder (Clarke and Youngstein, 2017). Our research findings suggest that pregabalin may be more strongly associated with withdrawal syndrome compared to gabapentin, corroborating evidence from multiple studies indicating pregabalin’s more significant addiction potential and its likelihood to cause withdrawal syndrome upon misuse (Bonnet and Scherbaum, 2017; Bonnet et al., 2018; Schifano et al., 2018; Vickers-Smith et al., 2020). It may be due to pregabalin’s higher binding affinity, more rapid absorption and higher bioavailability.

This study employs adverse event signal detection to compile a comprehensive list of drugs associated with withdrawal syndrome events. In clinical practice, mainly when prescribing medications for pain and mental health disorders, it is advisable to consider drugs with lower potential of withdrawal syndrome. Furthermore, it is imperative to implement targeted preventive measures, manage drug dosages carefully, and enhance monitoring to mitigate the risk of withdrawal reactions associated with these medications.

## 5 Conclusion

Through the analysis of the FAERS database, this study has compiled a comprehensive list of drugs associated with withdrawal syndrome, including the statistics of the most frequently reported drugs and their ROR signals. This information is invaluable for healthcare professionals, including doctors, pharmacists, and other health workers, as it enhances their ability to recognize and manage the adverse effects of drug withdrawal in patients. However, the study has its limitations. Disproportionality analysis only establishes a statistical association between drug usage and AEs and does not confirm a causal relationship between the drug and the adverse drug event (ADE) because the lack of details about patient exposure, reporting biases and confounding biases (Zhou et al., 2022; Li et al., 2023; Fusaroli et al., 2024). Moreover, there are phenomena such as underreporting, incomplete case information, duration of use and dosage cannot be considered when analyzing the association between drugs and AEs. Spontaneous reporting systems only record cases of drug-induced adverse events, it is not possible to accurately determine the total number of patients using these drugs (Noguchi et al., 2021). The analysis may also need to fully account for confounding factors that could influence the results, such as patients’ pre-existing medical conditions or concurrent medications. Additionally, while certain drugs, including other antidepressants, antipsychotic drugs, antiepileptic drugs, and central nervous system drugs, did not exhibit positive signals in this study, their clinical use still necessitates careful consideration and monitoring for potential withdrawal effects. Therefore, while the ADE signals highlighted in this study serve as a valuable reference, they should be interpreted with caution, and further research is needed to understand the complexities of drug withdrawal syndromes fully.

## Data Availability

Publicly available datasets were analyzed in this study. This data can be found here: http://www.fda.gov/drugs/surveillance/questions-and-answers-fdas-adverse-event-reporting-system-faers.

## Supplementary material

SUPPLEMENTARY TABLE S1 Top 50 medications associated with withdrawal syndrome from the FAERS arranged by ROR, 2004Q1 to 2023Q3.
