# Supplementary material for: Evaluating drug withdrawal syndrome risks through food and drug administration adverse event reporting system: a comprehensive disproportionality analysis
Source: Front Pharmacol. 2024 Jul 10;15:1385651. doi: 10.3389/fphar.2024.1385651 (PMC11266151; doi:10.3389/fphar.2024.1385651)
Supplement: Supplementary file 1 [file Table1.pdf]

Supplementary Table S1

Ranking	Medication	Classification	ROR	ROR lower bound, 95% CI	ROR upper bound, 95% CI	PRR	χ^2
1	Oxycodone	Opioids	79.51	78.46	80.58	72.88	1620308.56
2	Butorphanol	Opioids	48.72	42.10	56.38	45.15	8412.44
3	Hydromorhone Hydrochloride	Opioids	34.33	33.01	35.69	32.35	81318.65
4	Paroxetine	Antidepressant drugs	29.36	28.59	30.15	27.95	148399.43
5	Hydrocodone	Opioids	25.63	23.37	28.10	24.49	10692.12
6	Aspirin-Oxycodone Hydrochloride-Oxycodone Terephthalate Combination	Opioids	23.41	21.65	25.32	22.47	13409.48
7	Methadone	Opioids	21.18	19.94	22.49	20.41	20399.60
8	Buprenorphine Naloxone	Opioids	21.05	20.15	22.00	20.30	37638.44
9	Buprenorphine	Opioids	19.84	19.32	20.38	19.20	96718.78
10	Baclofen	muscle relaxant	18.49	17.64	19.38	17.91	28907.81
11	Duloxetine	Antidepressant drugs	17.41	17.00	17.83	16.92	103338.37
12	Naloxegol	Opioid antagonist drugs	13.23	11.15	15.69	12.97	1490.97
13	Morphine Naltrexone	Opioids	12.96	9.94	16.90	12.67	602.93
14	Hydrococone Acetaminophen	Opioids	11.03	10.24	11.88	10.82	6355.71
15	Venlafaxine	Antidepressant drugs	10.32	9.98	10.67	10.15	28520.12
16	Actemainophen Oxycodone	Opioids	7.94	7.39	8.54	7.84	4419.08
17	Morphine	Opioids	7.90	7.55	8.27	7.80	11135.17
18	Alprazolam	Antianxiety drugs	5.32	2.21	12.84	5.29	17.40
19	Fentanyl	Opioids	5.00	4.78	5.23	4.96	6027.54
20	Tapentadol	Opioids	4.15	3.43	5.02	4.12	253.40
21	Hyoscine	muscle relaxant	4.10	3.24	5.18	4.07	162.55
22	Tramadol	Opioids	3.37	3.12	3.63	3.35	1125.56
23	Lorazepam	Antianxiety drugs	3.09	2.76	3.46	3.08	428.24
24	Clonazepam	Antiepileptic drugs	3.04	2.78	3.33	3.03	631.81
25	Diazepam	Antianxiety drugs	2.71	2.40	3.06	2.70	282.02
26	Pregabilin	Analgesics	2.61	2.49	2.74	2.60	1706.46
27	Naltrexone	Opioid antagonist drugs	2.38	2.14	2.64	2.37	268.05
28	Ziprasidone	Antipsychotic drugs	2.10	1.68	2.63	2.10	44.32
29	Zolpidem	Hypnotic sedative drugs	2.10	1.86	2.38	2.10	146.67
30	Citalopram	Antidepressant drugs	1.97	1.82	2.14	1.97	278.75
31	Fluoxetine	Antidepressant drugs	1.91	1.70	2.14	1.90	121.36
32	Gabapentin	Antiepileptic drugs	1.76	1.63	1.90	1.76	223.32
33	Sertraline	Antidepressant drugs	1.63	1.47	1.81	1.63	88.61
34	Cetirizine	Anti-Allergic drugs	1.58	1.40	1.79	1.58	54.23
35	Loperamide	Antidiarrheals	1.58	1.31	1.91	1.58	23.16
36	Quetiapine	Antipsychotic drugs	1.54	1.44	1.66	1.54	143.30
37	Olanzapine	Antipsychotic drugs	0.96	0.86	1.07	0.96	0.52
38	Atomoxetine	Central nervous system drugs	0.93	0.76	1.13	0.93	0.54
39	Lisdexamfetamine Dimesylate	Central nervous system drugs	0.93	0.75	1.15	0.93	0.47
40	Varenicline	Central nervous system drugs	0.87	0.78	0.97	0.87	6.73
41	Aripiprazole	Antipsychotic drugs	0.63	0.56	0.72	0.63	52.12
42	Lamotrigine	Antiepileptic drugs	0.48	0.41	0.56	0.48	90.48
43	Levetiracetam	Antiepileptic drugs	0.44	0.36	0.54	0.44	69.76
44	Risperidone	Antipsychotic drugs	0.44	0.38	0.51	0.44	129.25
45	Sodium Oxybate	Narcotic drugs	0.39	0.34	0.46	0.40	151.07
46	Clozapine	Antipsychotic drugs	0.37	0.31	0.43	0.37	171.03
47	Mirtazapine	Antidepressant drugs	0.37	0.31	0.43	0.37	171.03
48	Natalizumab	immunosuppressive agents	0.14	0.11	0.17	0.14	494.64
49	Interferon Beta-1A	Anti-viral agent	0.08	0.06	0.11	0.08	579.49
50	Adalimumab	immunosuppressive agents	0.02	0.01	0.02	0.02	3478.39
